# Navigation of Pedicle Screws in the Thoracic Spine with a New Electromagnetic Navigation System: A Human Cadaver Study

**DOI:** 10.1155/2015/183586

**Published:** 2015-02-11

**Authors:** Patrick Hahn, Semih Oezdemir, Martin Komp, Athanasios Giannakopoulos, Richard Kasch, Harry Merk, Dieter Liermann, Georgios Godolias, Sebastian Ruetten

**Affiliations:** ^1^Center for Spine Surgery and Pain Therapy, Center for Orthopaedics and Traumatology of the St. Elisabeth Group–Catholic Hospitals Rhine-Ruhr, St. Anna Hospital Herne/Marienhospital Herne University Hospital/Marien Hospital Witten, Hospitalstrasse 19, 44649 Herne, Germany; ^2^Clinic for Orthopaedics and Orthopaedic Surgery, University Medicine of Greifswald, Ferdinand-Sauerbruch-Strasse, 17475 Greifswald, Germany; ^3^Institute for Diagnostic and Interventional Radiology and Nuclear Medicine, Marienhospital Herne, University Hospital of the Ruhr-University Bochum, St. Elisabeth Group–Catholic Hospitals Rhine-Ruhr, Hölkeskampring 40, 44625 Herne, Germany; ^4^Center for Orthopaedics and Traumatology of the St. Elisabeth Group–Catholic Hospitals Rhine-Ruhr, St. Anna Hospital Herne/Marienhospital Herne University Hospital/Marien Hospital Witten, Hospitalstrasse 19, 44649 Herne, Germany

## Abstract

*Introduction.* Posterior stabilization of the spine is a standard procedure in spinal surgery. In addition to the standard techniques, several new techniques have been developed. The objective of this cadaveric study was to examine the accuracy of a new electromagnetic navigation system for instrumentation of pedicle screws in the spine. *Material and Method.* Forty-eight pedicle screws were inserted in the thoracic spine of human cadavers using EMF navigation and instruments developed especially for electromagnetic navigation. The screw position was assessed postoperatively by a CT scan. *Results.* The screws were classified into 3 groups: grade 1 = ideal position; grade 2 = cortical penetration <2 mm; grade 3 = cortical penetration ≥2 mm. The initial evaluation of the system showed satisfied positioning for the thoracic spine; 37 of 48 screws (77.1%, 95% confidence interval [62.7%, 88%]) were classified as group 1 or 2. *Discussion.* The screw placement was satisfactory. The initial results show that there is room for improvement with some changes needed. The ease of use and short setup times should be pointed out. Instrumentation is achieved without restricting the operator's mobility during navigation. *Conclusion.* The results indicate a good placement technique for pedicle screws. Big advantages are the easy handling of the system.

## 1. Introduction

Stabilization of the spine using posterior pedicle screw instrumentation is a standard procedure in spine surgery [1–3]. The technique is used routinely for treating degenerative disorders, instability, deformities, fractures, and tumors. The correct positioning of the screws is one of the most important factors, as it ensures good pullout strength in the bone and good rotation and repositioning of the instrumented vertebral body [4]. Malposition of the pedicle screws can lead to serious complications such as injury to neural structures, blood vessels, and thoracic and intra-abdominal structures [5–7]. Radiculopathies, neuropathies, and hematomas typically occur as a result of malposition. The precise positioning of the screws is therefore essential. There are reports in literature of malpositions in 10–40% of cases and resulting revision rates of up to 6.6% [8, 9]. In addition to the exact selection of the entry point, the correct angle of trajectory, and consideration of anatomical variability in the different segments of the spine, preoperative imaging is recommended [10–12].

One of the standard techniques used today is pedicle screw insertion using fluoroscopic guidance in a lateral, anterior-posterior, or oblique ventrodorsal projection. These techniques are based on anatomical landmarks. In addition, intraoperative 3D imaging techniques (O-arm, 3D fluoroscopy, etc.) are also available today. The goal of all of these new developments is to increase the precision of screw placement [8, 13, 14]. Problems of existing techniques are increased intraoperative exposure to radiation using an intraoperative tomographic imaging technique (O-arm, 3D fluoroscopy) and increased malposition (free-hand placement) [15–17].

The navigation systems already available on the market should reduce malpositioning as well as reduce exposure of the operator, staff, and patients to radiation. The currently available navigation systems that are used in spine surgery can be classified as active or passive systems [18, 19]. There are also 3D-based techniques, in which navigation is based on a preoperative 3D data set. We differentiate between optoelectronic and electromagnetic navigation methods [20, 21]. Some of the problems with optoelectronic systems are that dynamic reference bases are required, to which active LEDs are attached that must have a continuous line of sight to the passive signal emitters (reflective spheres). This line of sight may never be interrupted, as this would impair navigation [22]. This may restrict the operator's normal range of movement and thus limit the intuitive handling of the instruments. The trackers needed for optical systems with active and passive reflectors are attached to the instruments and to the operation areas to be referenced and have anatomical and ergonomic disadvantages. For one thing, the instruments used are significantly larger and heavier, resulting in poorer ergonomics and handling for the operator. The trackers also protrude from the operation site so that they can be detected by the navigation camera. Only minimal changes in position due to unintentional contact to the reference base can lead to malpositions.

Unlike optoelectronic systems, electromagnetic navigation works with electromagnetic fields that penetrate the body through which navigation is controlled [23, 24]. This avoids the masking problems of conventional optical systems [25, 26]. Moreover, the reference bodies do not protrude from the body and their dimensions are minimized so that there is hardly any interference with the operator's normal workflow. The instruments are equipped with internal reference electrodes. Unlike other systems, they do not interfere with ergonomic movement, which has clear benefits with respect to the normal workflow.

The objective of this experimental cadaver study was to examine the precision of pedicle screw placement using a new electromagnetic navigation system (EMF navigation) on the human thoracic spine.

## 2. Material and Method

### 2.1. Ethics Statement

This study was conducted in compliance with the strict ethical guidelines for human cadaver studies. All body donors were fully legally competent and had a will in which they agreed to the use of their body or body parts for research, study, or teaching purposes (Section for Clinical Anatomy, Heinrich Heine University, Düsseldorf, Germany). The ethics committee of the Medical Association of Westfalen-Lippe gave its approval for this study (214-037-f-S).

### 2.2. Bodies Used

The study was conducted on human bodies with intact spines that were preserved using the Thiel method (Section for Clinical Anatomy, Heinrich Heine University, Düsseldorf, Germany). All bodies were examined in advance by a thin-slice CT scan to check for previous operations on the spine, pathological changes, tumors, and severe anomalies. A total of 48 pedicle screws were inserted in the thoracic spine using EMF navigation.

### 2.3. EMF Navigation System

A new EMF navigation system with clinical approval for ENT and neurosurgery (Fiagon GmbH, Hennigsdorf, Germany) and adapted for spine surgery was tested. Special instruments were developed (“CenterPointer”, “AwlPointer”, “SpinePointer,” and a navigable screwdriver) to make navigation in the EM field possible. The technique of this new electromagnetic navigation system is based on continuous tracking of the instruments and the patient's anatomical structures in three-dimensional space during the entire surgical procedure. The system provides the operator with additional information without changing or affecting the normal standard procedure. The system can correct static errors using special calibration and correction methods. It takes errors into account that are attributable to the position and alignment of the navigating instrument. This leads to improved position precision in the working area adapted for the application. Navigation is carried out virtually, in real time, in a 3D data set. An exact intraoperative assessment is therefore possible in all planes.

### 2.4. Tracking System and Instruments Developed

**Figure 1:**
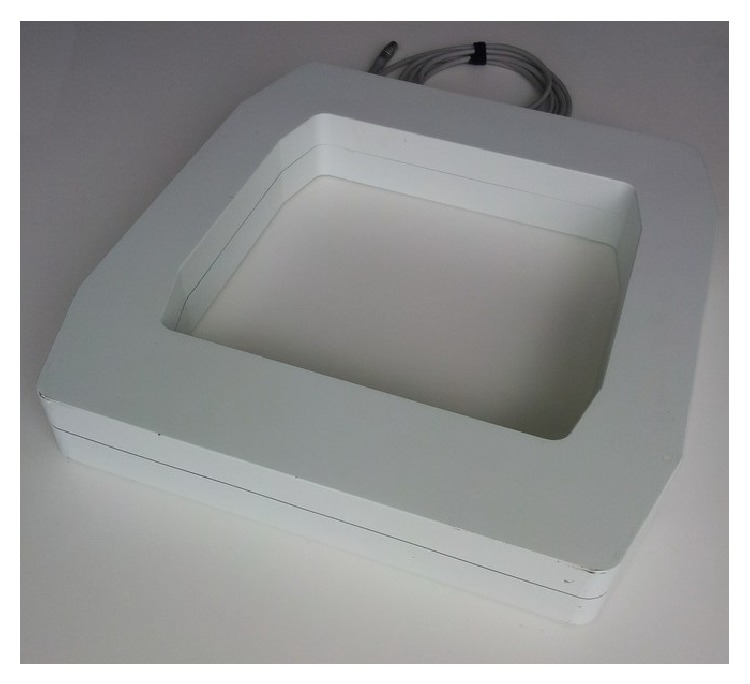
Field generator.

For navigation an electromagnetic field is generated that allows the instruments in the area to be detected. A special field generator (Figure 1) is used for this. In this study the field generator was placed under the Patient. The frame enclosed the entire surgical field. In the final version the field generator will be integrated to the operating table. The instruments used are equipped with specially produced signal coils positioned inside the instruments. For matching the anatomical structures, image data are used that were generated preoperatively in a thin-slice CT scan. For the tracker, a so-called patient tracker (Figure 3) was attached to the spinous process of the vertebral body to be instrumented. This tracker can be detected in the magnetic field.

Specially developed instruments were used that are adapted to standard instruments used today. The instruments have special hollow spaces that can hold the necessary navigation coils. These navigation coils are firmly anchored with the instruments, so standard sterilization can be carried out.

**Figure 2:**
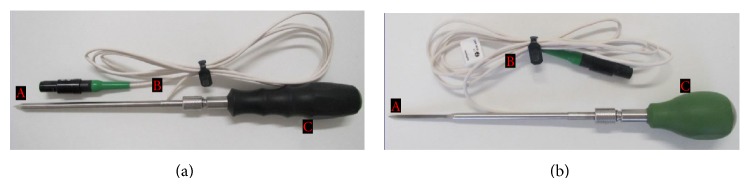
Surgical instruments with integrated electromagnetic field sensor, (a) CenterPointer, (b) AwlPointer; A: navigation of the tool tip, B: cable, and C: handle.

**Figure 3:**
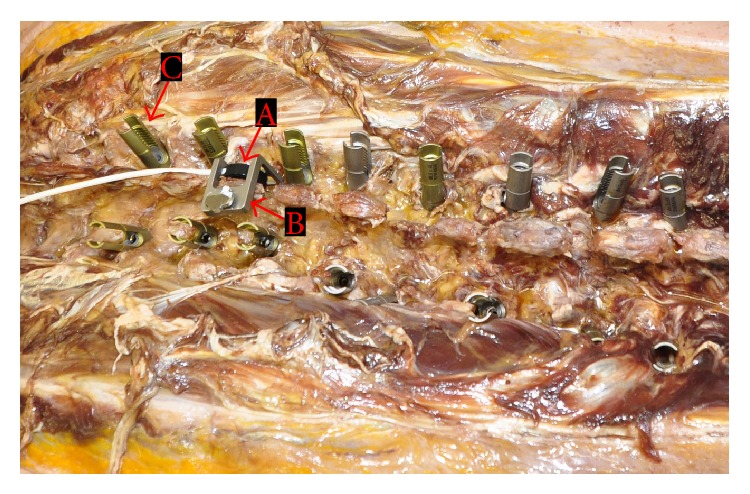
Cadaver with patient tracker (A), fixation clamp (B), and inserted screws (C).

For navigation, a CenterPointer, AwlPointer, SpinePointer, and a special navigable screwdriver were used (Figure 2). All instruments used are comparable with the standard instruments for spine surgery. The CenterPointer was used for the required surface matching and to open the pedicle. The AwlPointer was used to open the vertebral body in the corresponding trajectory and to determine the screw length needed. The SpinePointer was used to probe the pedicle in order to detect and visualize malpositions and injuries to the surrounding cortical bone.

All instruments are connected with the navigation system by a wire.

All pedicle screws used were polyaxial screws with a diameter of 4.5–6 mm, depending on the pedicle width measured in the vertebral body to be navigated (S4-System, Braun Melsungen, Germany).

### 2.5. Preoperative Planning

A CT scan with a 1 mm slice thickness was made of all specimens required and a 3D reconstruction was generated (Institute for Diagnostic and Interventional Radiology, Düsseldorf University Hospital, Germany). The data set was used to plan the navigation; for this the diameter of the respective pedicle was calculated and trajectory was determined. For further intraoperative processing and navigation, the data set was imported to the navigation system in DICOM format. From this data set, a high-resolution three-dimensional VRT (volume-rendered tomography) model was calculated by the navigation system that was used for navigation. The system simultaneously calculated another 3D data set for navigation in all planes.

### 2.6. Navigation

All cadavers were placed in prone position on a special, nonmetallic carbon operating table (MAQUET Holding GmbH & Co. KG, Rastatt, Germany) to prevent interference from metallic objects. Surgery was performed via a standard approach with a midline incision and standard preparation. Following this, the patient tracker was attached to the spinous process to form the reference to the navigation unit in the coordination system. The tracker was attached to the spinous process of each vertebral body to be instrumented. Then surface matching of the vertebral body was carried out using the CenterPointer for a total of 20 freely selected points. This is done to match the data set with the electromagnetic coordinate system. After performing an error calculation in the system and releasing it for navigation, the operator made an optical check using anatomical landmarks. Navigation took place only after the optical check for errors. The system offers a direct interaction with preoperative DICOM images in all spatial planes.

The AwlPointer is used to determine the insertion point, drill a pilot hole, and open the pedicle. Particular attention was paid to the precise position of the trajectory to the pedicle. A software tool was used to virtually calculate the length and diameter of the pedicle screws. In addition, the CenterPointer was used for a tactile probe of the pedicle and vertebral body (5 corticalices) followed by the controlled navigated insertion of the screw under pure navigation without fluoroscopy guidance. The procedure is visualized in real time in the navigation system and the operator has the option of checking the position in all spatial planes. Visualization can be adapted as needed.

### 2.7. Follow-Up

After the study, the cadavers were again examined by CT scan and evaluated.

Grade 1: ideal screw position in the center of the pedicle with no injury to cortical bone, grade 2: acceptable screw position, cortical bone injury with maximum penetration of 2 mm, and grade 3: cortical bone injury with penetration >2 mm.

A statistical analysis was made using an analysis of confidence intervals.

## 3. Results and Discussion

A total of 48 pedicle screws were evaluated. The minimum width of thoracic pedicles was 4.8 mm (TH3); the maximum width was 8 mm (Th12). The median pedicle width was 5.9 mm with a standard deviation of 0.76 (Table 1). The diameter of the pedicle screws used was adapted to the pedicle width. All screws were polyaxial screws.

20 screws had a grade 1 position, 17 screws had an acceptable screw position (grade 2), and 11 screws a malposition (grade 3). The initial evaluation of the system showed a satisfied positioning for the thoracic spine. There was thus a maximum pedicle perforation of no more than 2 mm in 77% of all pedicle screws placed. The 95% confidence interval for the success rate of the screws in the thoracic spine was 62.7% (88.0%). These were the screws classified as grade 1 or 2. Malposition occurred in most cases (9 screws) in pedicles with the highest ratio (Th 3, Th 2, Th 9).

Posterior instrumentation using pedicle screws is a standard technique in spine surgery [27]. In addition to free-hand positioning and fluoroscopy-guided techniques, several different tomographic imaging techniques (3D fluoroscopy, cone beam CT, intraoperative CT scan) have contributed to greater intraoperative precision in positioning pedicle screws [28–30]. Additionally, several different navigation systems are used. The aim of all these techniques is to achieve the greatest precision in inserting the pedicle screws.

The objective of this study was the preclinical evaluation of a new EMF navigation system for positioning pedicle screws in the thoracic spine [31–33]. The navigation of the pedicle screws was conducted using an EMF navigation system modified for the thoracic spine. Navigation was based on a CT data set that had been calculated prior to the intervention.

The method of EMF navigation of pedicle screws based on a preoperative CT data set has not yet been described in literature.

One advantage of this method is the intraoperative virtual visualization in real time that is a clear advantage over the 2D visualization using a fluoroscopy data set. The system is also easy to handle and uses standard instruments that are nearly unmodified. Unlike optical navigation systems, there are no optical reference spheres outside the site or on the instruments that can interfere with the operator's normal workflow or lead to miscalculations in the system if they are accidentally contacted.

There is nearly no intraoperative exposure to radiation when navigation is carried out only using this new EMF technique. However, a preoperative thin-slice CT data set is necessary, which may result in an increase of the total radiation dose for the patient. The evaluation of the radiation exposure must therefore be investigated in further clinical studies.

The reference coils used in the instruments are not yet located right at the tip of the instrument, so torsion forces from deflection can lead to the malpositions that were measured in this study. This particularly affects small pedicle diameters that have only a low margin of error.

In addition, the analysis and the CT measurement showed a relative mobility of the polyaxial screws (shaft/head) of about 7°, and the higher margin of error can also be caused by this.

## 4. Conclusion

The analysis of the pedicle screw positions in the thoracic spine showed satisfied precision but was not yet clearly superior to other systems. In comparison with the lumbar spine that has larger pedicle sizes compared to pedicle screw diameter, the thoracic spine has a lower margin of error.

In summary, the results indicate a good placement technique for pedicle screws in the thoracic spine, although modifications of instruments and the screwdriver-screw interface must be improved to reduce deviation tolerances. Additional studies with larger populations and smaller pedicle diameters, for example, dysplastic pedicles like neuromuscular scoliosis, should be conducted. Big advantages are the easy handling of the system, the intraoperative matching, and the nearly unchanged surgical procedure for the operator. The reduction of intraoperative exposure to radiation for the operator and the entire surgical team is another advantage over existing systems.

## Figures and Tables

**Table 1:** Relation screw diameter to pedicle diameter.

	Pedicle diameter [mm]		Screw diameter [mm]	Ratio %
	Mean	Stand.-dev.		
Th1	6,9	0,2	5	72,5
Th2	5,5	0,7	5	90,1
Th3	4,8	0,1	4,5	94,8
Th4	5	0,2	4,5	90
Th5	5,3	0,7	4,5	84,9
Th6	5,9	0,5	5	85,8
Th7	5,8	0,3	5	56,2
Th8	5,7	0,1	5	87,7
Th9	6,2	0,3	5	80,6
Th10	6,4	1	5	78,1
Th11	6,3	0,4	5	89,4
Th12	7,4	0,6	6	81,1
